# iTRAQ-Based Quantitative Proteomics and Transcriptomics Provide Insights Into the Importance of Expansins During Root Development in Carrot

**DOI:** 10.3389/fgene.2019.00247

**Published:** 2019-03-29

**Authors:** Ya-Hui Wang, Feng Que, Guang-Long Wang, Jian-Nan Hao, Tong Li, Zhi-Sheng Xu, Ai-Sheng Xiong

**Affiliations:** ^1^State Key Laboratory of Crop Genetics and Germplasm Enhancement, Ministry of Agriculture and Rural Affairs Key Laboratory of Biology and Germplasm Enhancement of Horticultural Crops in East China, College of Horticulture, Nanjing Agricultural University, Nanjing, China; ^2^School of Life Sciences and Food Engineering, Huaiyin Institute of Technology, Huai’an, China

**Keywords:** *Daucus carota*, expansins, expression profiles, proteomics, root formation and enlargement

## Abstract

Carrot is an important root vegetable crop with a variety of nutrients. As the main product of carrots, the growth and development of fleshy roots directly determine the yield and quality of carrots. However, molecular mechanism underlying the carrot root formation and expansion is still limited. In our study, isobaric tags for relative and absolute quantification (iTRAQ) was utilized to explore the differentially expressed proteins (DEPs) during different developmental stages of carrot roots. Overall, 2,845 proteins were detected, of which 118 were significantly expressed in all three stages. DEPs that participated in several growth metabolisms were identified, including energy metabolism, defense metabolism, cell growth and shape regulation. Among them, two expansin proteins were obtained. A total of 30 expansin genes were identified based on the carrot genome database. Structure analysis showed that carrot expansin gene family was relatively conserved. Based on the expression analysis, we found that the expression profile of expansins genes was up-regulated during the vigorous growing period of carrot root. Furthermore, there was a consistent relationship between the expression patterns of mRNA and protein. The results indicated that expansin proteins might play important roles during root development in carrot. Our work provided useful information for understanding molecular mechanism of carrot root development.

## Introduction

Carrot (*Daucus carota* L.) is one of the most economically important horticultural crops worldwide. In 2017, the Food and Agriculture Organization (FAO) of the United Nations estimated that the world’s gross cultivated area of carrots was about 1.14 million ha with carrot production of China occupying the first place all over the world. In the food industry, carrot roots are commonly commercialized raw as a fresh vegetable, or are processed mainly as bioethanol resources and salads (Iorizzo et al., 2011; Aimaretti et al., 2012). Carrot taproot is a good source of nutrients, such as carotenes, anthocyanins and vitamins (Yildiz et al., 2013; Luby et al., 2014; Perrin et al., 2017). Fleshy root is the main product organ of carrots, its growth and development determines the yield and quality of carrots directly. The formation and enlargement of carrot root is a complex process that involves gene expression, protein functions, structural changes, and matter accumulation (Xu et al., 2014; Li et al., 2015).

Nowadays, proteomic technology has become an efficient tool to understand the protein composition within plants and their dynamic changes under specific conditions (Li et al., 2015, 2018; Cao et al., 2017). The most classic technique for protein separation is the two-dimensional gel electrophoresis (2-DE), whereas differentially expressed spots are observed and analyzed by mass spectrometry (MS). Recently, isobaric tags for relative and absolute quantitation (iTRAQ) labeling technology has been demonstrated to provide relative quantification for thousands of proteins at the same time. iTRAQ technique possesses higher accuracy and sensitivity, which is thus widely used for protein separation and analysis (Aggarwal et al., 2006; Zieske, 2006). Proteomic responses during the development of a particular plant organ/tissue have been identified in various crops, such as rice, potato, castor, grape, and tea plant (Ma et al., 2012; Martinez-Esteso et al., 2013; Nogueira et al., 2013; Li et al., 2015; Liu et al., 2017; Wu et al., 2018). The results of these studies provided a large number of DEPs during plant development. However, proteins responding to developmental processes varied considerably in plant species and tissues. Especially in carrots, proteome information related to root growth and development is still limited.

Expansin is a kind of wall-loosening protein which can be found in almost all plant species (Cosgrove, 2000a,b). In the 1990s, it was first identified in cucumber (*Cucumis sativus*) (McQueen-Mason et al., 1992; Simon and Daniel, 1994). According to the phylogenetic sequence analysis, the expansin superfamily was divided into four subfamilies named as EXPA, EXPB, EXLA, and EXLB (Sampedro and Cosgrove, 2005). Previous functional research has demonstrated that expansins could involve in several plant growth and development processes, such as plant cell growth (Cosgrove et al., 2002), xylem formation (Gray-Mitsumune et al., 2004), seed germination (Chen and Bradford, 2000), fruit ripening (Harrison et al., 2001) and some other developmental processes. Therefore, it is advisable to conduct a systematic analysis of expansins to provide a basis for further exploring the molecular mechanism of carrot growth and development.

In this study, the DEPs and their dynamic changes during carrot root development were analyzed by iTRAQ technology. Among them, two expansin proteins were identified. The expansin gene family in carrot genome was further explored. We also analyzed the expression profile of *DcEXP* genes by combining the data of transcriptome and RT-qPCR experiments. Our results are expected to facilitate deep insights into the carrot expansin gene family and provide an improved understanding of the molecular mechanism underlying carrot root development.

## Materials and Methods

### Plant Materials

The carrot cultivar, ‘Kurodagosun’, was used as experimental material in this study. ‘Kurodagosun’ originates from Japan and is commonly cultivated in China. It has an orange root with high and stable yield and good quality (Xu et al., 2014). Here, carrot seeds were sown in pots filled with a mixture of organic soil and vermiculite (v/v, 1:1). Carrot plants were grown in a growth chamber with a 14 h/10 h light/dark regime. Growth temperature was set as 25 or 18°C during daytime or nighttime, and the relative humidity was kept as 60–70%. Whole roots of carrot at 20, 40, and 90 DAS were collected and immediately immersed in liquid nitrogen. Three biological replicates were performed at each stage. Each biological replicate consisted of three individual seedlings. Then, the samples were allowed to store at −80°C until further analysis.

### Histochemical Staining

Approximately the middle parts of the fresh carrot roots obtained at three developmental stages were cut into around 2 mm-thick slices for anatomical structure analysis. Samples were stored in formalin-aceto-alcohol (38% formaldehyde: glacial acetic acid: 50% ethanol = 1:1:18, v/v/v) for subsequently safranin-*O*/fast green staining. First, we deparaffinized the root samples in xylene and then dehydrated them by ethanol. The staining process was performed as follows: the dehydrated sections were stained in 1% safranin-*O* for 2 h, and then counterstained by 0.5% fast green for 15 s, 95% ethanol was utilized to remove excess stain finally.

### Protein Extraction

Total protein was extracted using the Nito Extra^TM^ kit (Cat. PEX-001-250ML, N-Cell Technology). Tissue debris was removed by high speed centrifugation at 28,000 rpm for 2 h at 10°C. Protein was precipitated with cold acetone at −20°C overnight. Subsequently, the sediment was air dried and washed twice with cold acetone then resuspended in 8 M urea.

Protein in solution was precipitated by adding three volumes of pre-cooled acetone. White protein precipitates were observed upon mixing. The mixture was incubated at −20°C for 12 h to maximize the efficiency of protein precipitation. Precipitated proteins were then harvested by centrifuging the mixture at 14,000 rpm for 15 min. The protein pellet was washed twice with equal volume of pre-cooled acetone. The acetone remaining in the protein was removed by evaporation.

The protein pellets were directly resuspended in 8 M urea. Samples were reduced with 20 mM DTT at 60°C for 1 h and alkylated with 40 mM indole acetic acid (IAA) in the dark at room temperature for 30 min. Alkylation reaction was quenched by 10 mM DTT. The urea concentration was diluted to 2 M urea with HPLC grade water. Trypsin was then added to the sample with an enzyme-to-substrate ratio of 1:100, and the digestion was performed in 100 mM TEAB at 37°C for 18 h. Digested proteins were desalted with C_18_ ZipTip (Millipore) for LC-MS/MS analysis.

### iTRAQ Labeling

Approximately 100 μg of desalted peptides was chemically labeled with iTRAQ 8-plex reagent in 100 mM TEAB according to the manufacturer’s instructions. In brief, isopropanol was added to each iTRAQ reagent tube to make sure that there was at least 70% of isopropanol existing in the tube. The pH of combined sample was checked again to ensure that the final pH was between 7 and 10. The labeling reaction was allowed to carry out at room temperature for 2 h. All of the labeled samples were then dried in spin for C_18_ desalting.

### LC-MS/MS Analysis

Each dried peptide sample was dissolved in 12 μL of 0.1% FA. The sample was quantified by nano LC-MS/MS using an Eksigent ekspert^TM^ nano LC 425 system coupled with an AB Sciex TripleTOF^^®^^ 6600 system. Peptides were sampled on a 350 μm × 0.5 mm trap column and eluted into a 75 μm × 15 cm C_18_ column using a linear gradient of acetonitrile (3–36%) in 0.1% FA at a flow rate of 300 nL ⋅ min^−1^. Mass spectra and tandem mass spectra data were acquired in positive-ion and ‘high-sensitivity’ mode with a resolution of ∼35,000 full-width half-maximum. The nano spray needle voltage was typically 2,300 V in HPLC-MS mode. For collision induced dissociation tandem mass spectrometry, the mass window for precursor ion selection of the quadrupole mass analyzer was m/z ± 2. The precursor ions were fragmented in a collision cell using nitrogen as the collision gas. Advanced information dependent acquisition was applied for MS/MS collection to obtain MS/MS spectra for the 20 product ions following each survey MS1 scan over a 250 ms acquisition time per MS/MS experiment. Exclusion duration was set for 30 s after two repetitive occurrences. The MS proteomics data have been deposited to the ProteomeXchange Consortium^1^ *via* the iProX partner repository with the dataset identifier PXD011983 (Ma et al., 2018).

### Standard Mascot Search

Raw data was analyzed with Mascot Daemon software (Version 2.5, Matrix Science). Peptide mass tolerance was set as 50 ppm and 0.05 Da. Carbamidomethylation (+57 Da) was set as a fixed modification, while oxidation (Specificity: M, Delta: +16 Da) was added as variable modifications. Peptides were assumed to have a charge of 2+, 3+, or 4+, allowing for two missed cleavages. FDR was controlled under 1% with Decoy database.

### Bioinformatics and Annotations

BLAST analysis was carried out using blastp developed by NCBI against the ‘non-redundant protein sequences (nr)’ database, where the matrix BLOSUM62 was used. The gene ontology (GO) annotations and Enzyme Commission numbers were then assigned to the proteins using BLAST2GO plugin of CLC workbench according to the results of BLAST analysis. Fisher’s exact test was applied to compare the distribution of differential proteins with that of all detected proteins in GO categories. As a result, the enriched GO categories were filtered by FDR = 5%.

### Identification of Expansin Genes in Carrot

The 36 nucleotide sequences of *Arabidopsi*s expansins obtained from ‘The *Arabidopsis* Information Resource database’^2^ were used as basic queries against the genome sequences of carrot in the ‘Phytozome database’^3^. By using ‘Superfamily online website’^4^, the optional *DcEXP* gene sequences were examined to ensure the presence of the expansin-specific domains. DNAMAN 6.0 was used to compare the amino acid sequences of these expansins. Phylogenetic trees were generated by MEGA 5.0 (Tamura et al., 2011). MEME was applied to identify the motifs of paralogous expansin proteins (Bailey et al., 2009). Theoretical isoelectric point (*pI*) and molecular weight (Mw) of the proteins were calculated by ‘The Sequence Manipulation Suite’^5^ (Stothard, 2000). We also predicted the signal peptide by the SignalP 4.1 server^6^. HemI was used to draw the dendrogram of DEPs and heat map that indicated the transcript levels (Deng et al., 2014).

### RNA Extraction and cDNA Synthesis

Total RNA was extracted from carrot taproots using a plant RNA extraction kit (Tiangen, Beijing, China) according to the manufacturer’s instructions. Concentration of each RNA sample was detected by One-Drop microvolume spectrophotometers. 1,000 ng of the total RNA was used for first-strand cDNA synthesis using PrimeScript RT reagent kit (TaKaRa, Dalian, China) in accordance with the manufacturer’s protocol.

### Gene Cloning and Expression Validation of Differentially Expressed Expansins

Based on the carrot genomic and transcriptomic database (Xu et al., 2014; Iorizzo et al., 2016), two expansin genes (*DcEXP20*, GenBank No. MK190680; *DcEXP22*, GenBank No. MK190681) were cloned by reverse transcription-polymerase chain reaction (RT-PCR). The reaction condition was set as follows: 94°C for 5 min; followed by 35 cycles of 94°C for 30 s, 54°C for 30 s, 72°C for 1 min; 72°C for 10 min. The amplification product was separated using 1.2% agarose gel electrophoresis, and then inserted into the pMD19-T simple vector (Takara, Dalian, China). After transformed into the *Escherichia coli* strain (DH5α), the product was sequenced by Genscript Inc. (Nanjing, China). Quantitative RT-PCR (RT-qPCR) was applied to validate the expression levels of expansins during carrot root development. The reaction was performed according to the following conditions: 95°C for 30 s, 40 cycles of 95°C for 5 s and 60°C for 30 s. A melting curve was used to check the specificity of each amplification, whose temperature was increased from 65 to 95°C at a rate of 0.5°C every 10 s. Relative gene expression levels were calculated based on the cycle threshold (*C*t) values and normalized against the carrot reference gene *DcActin* (Wang et al., 2016). The primer sequences for cloning and RT-qPCR were listed in Supplementary Table S1, Supplementary Material S1. Normalized *C*t values were used for the Pearson correlation coefficient analysis with the help of SPSS software.

### Statistical Analysis

One-way ANOVA was used to statistically analyze the data with the help of SPSS version 20.0. Significant differences were analyzed on the basis of Duncan’s multiple range test at 0.05 probability.

## Results

### Plant Growth Analysis

**FIGURE 1:**
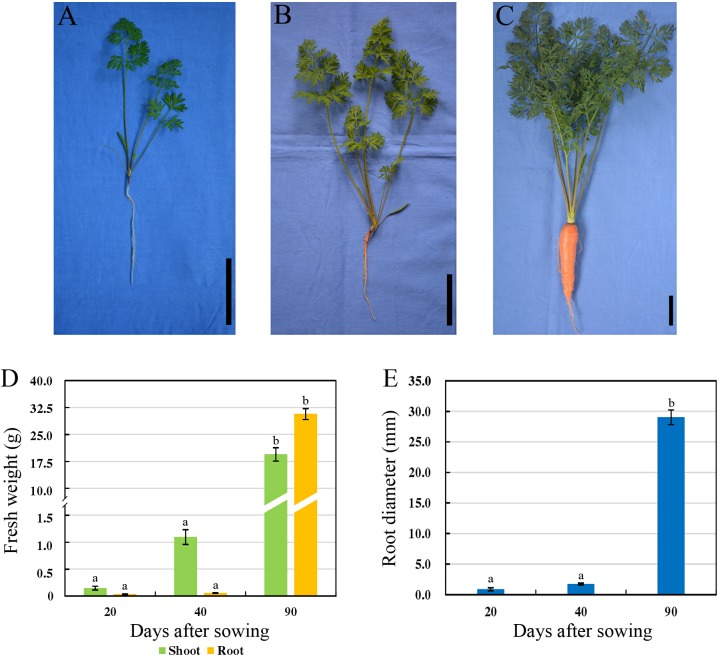
Morphological characteristics of carrots at three developmental stages. **(A–C)** Growth status of carrot ‘Karodagosun’ at 20 DAS **(A)**, 40 DAS **(B)**, and 90 DAS **(C)**. The black lines in the right lower corner in each image represent 5 cm. **(D)** Fresh weight of carrot roots at three developmental stages. **(E)** Root diameter of carrots at three developmental stages. Different lowercase letters indicate significant differences at *P* < 0.05.

Carrot roots at the seedling stage (20 DAS, S1), breaker stage (40 DAS, S2), and mature stage (90 DAS, S3) were harvested, respectively (Figure 1A–C). At 20 DAS, carrot root was white, and the fresh root weight was obviously lower than the shoot weight (Figure 1D). At breaker stage, carrot root began to show orange color on the surface, and the fresh shoot weight was improved greatly. During this stage, neither the fresh root weight nor diameter showed significant changes (Figure 1E). In the succeeding period of time, the root grew fast, and the fresh weight of taproot was higher than that of shoot (Figure 1D). Meanwhile, the root diameter was significantly increased during carrot root growth (Figure 1E).

**FIGURE 2:**
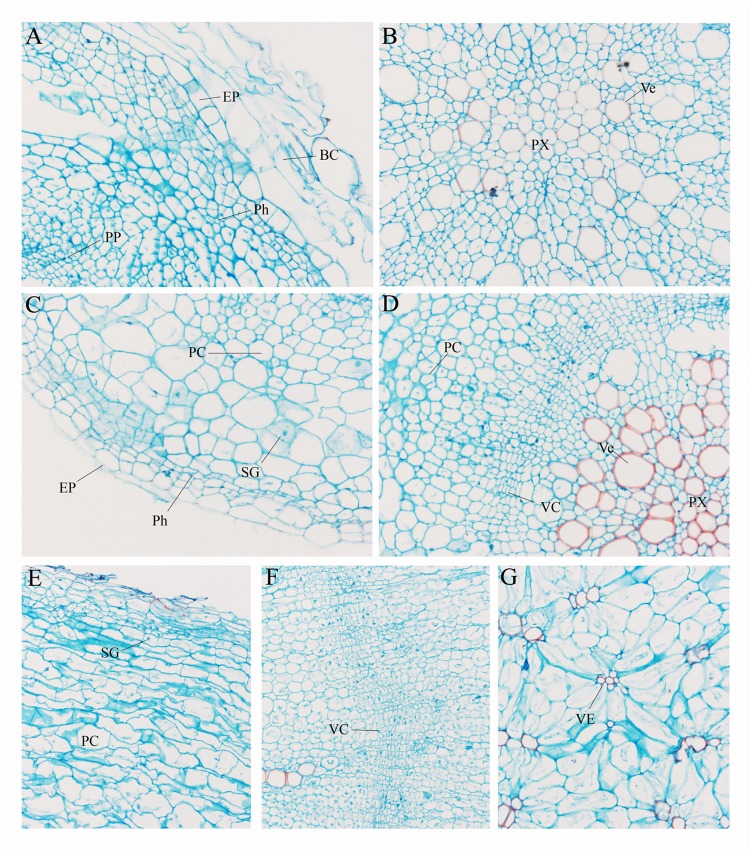
Anatomical structure of carrot roots from Stage 1 **(A,B)**, Stage 2 **(C,D)** and Stage 3 **(E–G)**. BC, border cells; EP, epidermis; PC, parenchymal cell; Ph, phellogen; PP, primary phloem; Px, protoxylem; SG, starch granule; VC, vascular cambium; Ve, vessel. The magnification is 200 times the original size in **(A–D)** whereas the magnification is 400 times in **(E–G)**.

To further explore the anatomical changes during carrot root development, Safranin-*O*/fast green staining was employed to display the structure of different plant tissues (Figure 2). In the presence of staining, lignified cell walls can turn red. As shown in Figure 2A, there were quantities of large-sized border cells during the seedling stage. At this stage, the phloem and protoxylem were small and the lignification was not obvious (Figure 2B). With the increase in number and size of these cells, the carrot roots began to thicken. At the second stage, the vascular cambium divided constantly. Lots of vessels were closely arranged in the xylem, and the lignification of cell wall was significantly increased (Figure 2C,D). Hereafter, at the later stage of root development (Figure 2E–G), distribution of the vessels in xylem changed greatly, which began to spread out in clusters instead of arranging closely. Meanwhile, a large number of starch granules accumulated rapidly during this period.

### Protein Identification and Quantification

**FIGURE 3:**
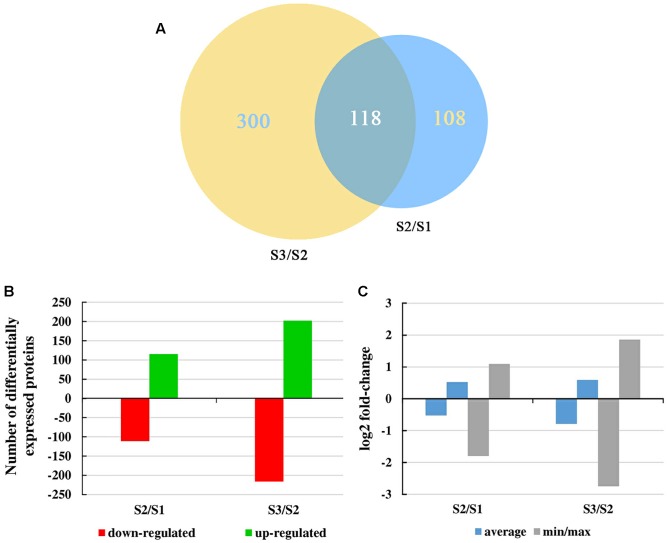
Overall changes in the protein level throughout carrot root development. **(A)** Venn diagram showing the number of proteins commonly and uniquely expressed at different developmental stages. Numbers in an oval denote phase-specific proteins, and number in two intersecting ovals represents overlapped proteins. **(B)** Number of differentially expressed proteins between two consecutive developmental stages. **(C)** Mean and max/min changes of the differentially expressed protein log2fold-change between two consecutive developmental stages.

iTRAQ technology was used to analyze proteins obtained from carrot roots at the adjacent stages. Overall, 2,845 proteins were detected. Over the period of carrot root growth, a total of 226 and 418 DEPs were identified at the breaker stage (S2) and mature stage (S3), respectively (Supplementary Material S2). Moreover, 118 DEPs were simultaneously present in the two stages (Figure 3A).

Figure 3B displayed the number of up- and down-regulated proteins during carrot root growth. In each phase, the number of up- and down-regulated proteins were similar. However, the number of DEPs at the mature stage was almost twice of that at the breaker stage. Meanwhile, the average and the minimum/maximum value of the DEPs were illustrated in Figure 3C. During the mature stage, the changing amplitude of the DEPs was relatively more pronounced.

### Classification of Metabolic Process-Specific Proteins

Based on the GO database, we conducted a functional annotation of all the DEPs between two consecutive developmental stages. The top 20 GO terms of three categories ‘biological process,’ ‘cellular components,’ and ‘molecular function’ in accordance with their *P*-values were displayed in Supplementary Figure S1, Supplementary Material S1. Most of the DEPs were enriched in the group ‘cellular components’ both in the two sets (S2/S1, S3/S2). Meanwhile, the great majority of the GO terms in this group were quite closely. As an example, the ‘nucleus’ and ‘nucleolus’ were the most represented GO terms in both S2/S1 (63 and 55 DEPs, respectively) and S3/S2 (91 and 79 DEPs, respectively). The same phenomenon occurred in the ‘molecular function’. For ‘biological process,’ there were some differences between the GO terms of the two sets. The ‘response to water deprivation’ and ‘nucleosome assembly’ contained the most DEPs in S2/S1 (19 DEPs) and S3/S2 (28 DEPs), respectively.

**FIGURE 4:**
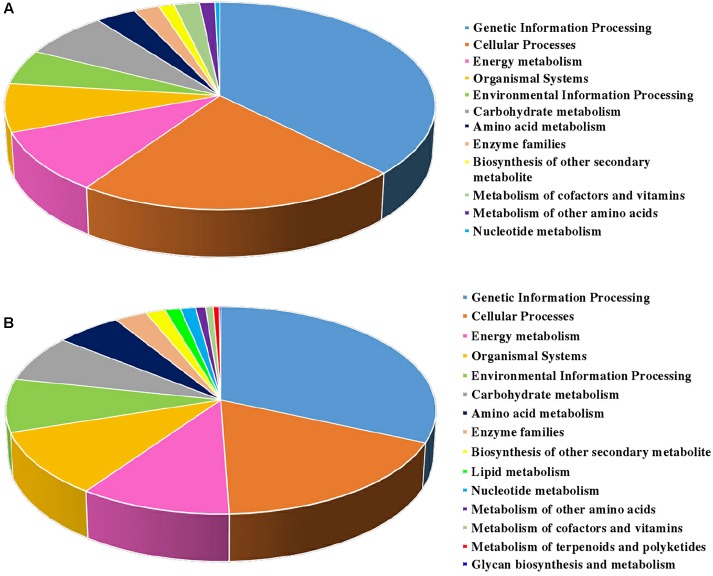
KEGG classification of DEPs identified from carrot roots at different developmental stages. **(A)** KEGG classification of DEPs identified between S1 and S2. **(B)** KEGG classification of DEPs identified between S2 and S3.

The biological pathways were also analyzed by utilizing the KEGG database (Figure 4). A total of twelve pathways were consistent in the two sets. Among them, ‘genetic information processing,’ ‘cellular process,’ ‘energy metabolism,’ and ‘organismal systems’ took the largest proportions of all the pathways in S2/S1 (37.4, 22.3, 9.7, and 7.6%, respectively) and S3/S2 (31.6, 17.9, 10.4, and 10.2%, respectively). The ‘lipid metabolism’, ‘metabolism of terpenoids and polyketides’ and ‘glycan biosynthesis and metabolism’ pathways were specially found in S3/S2.

### Profiles of the Proteins’ Functional Clusters During Carrot Root Development

**FIGURE 5:**
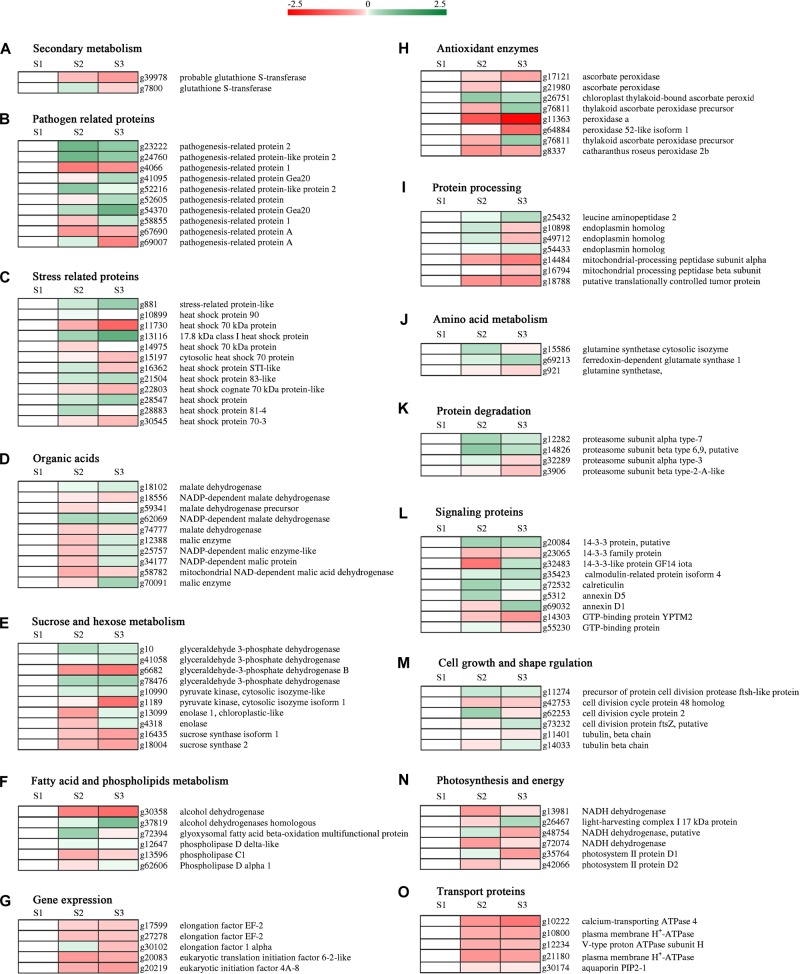
Hierarchical clustering analysis of protein expression at different developmental stages. Heat maps were created by the log2 relative abundance of proteins. The accession number and protein description for each protein were exhibited. Proteins were grouped according to their roles in metabolic pathways. **(A–O)** Represent different protein functions.

Carrot tissue proteome determines the growth and development of fleshy root. Different expression levels of various proteins during carrot root development underlines special function and importance at different phases. As shown in Figure 5, some of the DEPs were clustered according to their putative biological functions. During carrot root development, expression levels of several pathogen related (Figure 5B), stress related (Figure 5C), protein degradation related (Figure 5K) and signaling proteins (Figure 5L) continued to improve. As for proteins involved in organic acid metabolism (Figure 5D), the majority of these DEPs were downregulated at S2. On the contrary, the expression levels showed increase at maturity. The results also showed the decrease in expression of elongation factors (Figure 5G), antioxidant enzymes (Figure 5H), photosynthesis and energy related proteins (Figure 5N) and transport proteins (Figure 5O). See as a whole, different proteins with the same function expressed different trends.

### Identification of the Expansin Proteins in Carrot

Based on protein annotation, two expansin proteins were obtained from the proteomics data of carrot roots during different developmental stages. Information including *pI*, protein Mw and so on was listed in Table 1. The expression levels of these two expansin proteins were similar, which rose first and then declined.

**Table 1:** Annotation and expression of identified expansin proteins.

Accession number	Protein description	Protein *pI*	Protein Mw/Da	Total peptides	Protein coverage/%	Geometric ratio	
						S2/S1	S3/S2
g13058	Expansin-B3-like	9.198	28188.3	10	12.261	1.4697	0.7450
g63815	Beta-expansin 3	8.557	35026.2	4	6.607	1.3785	0.7312

In order to identify the genes of these two expansin proteins, cloning primers were designed in accordance with the protein sequences. Two *DcEXP* genes, *DcEXP20* and *DcEXP22* were cloned by RT-PCR and their open reading frames (ORF) were 786 bp and 789 bp, encoding 261 and 262 amino acids, respectively (Supplementary Figure S2, Supplementary Material S1).

**FIGURE 6:**
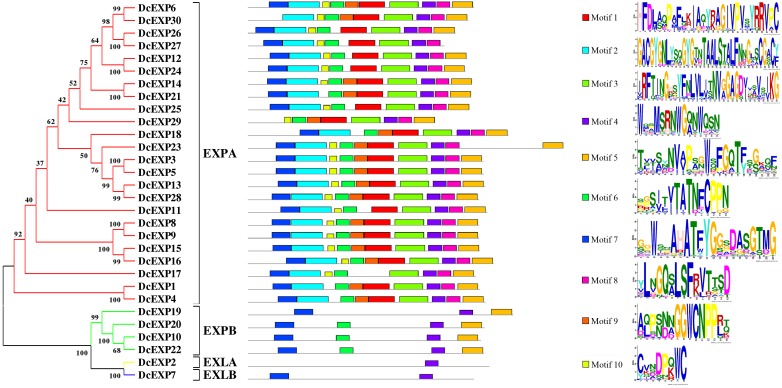
Phylogenetic tree and motif compositions of carrot expansin genes.

To make a thorough inquiry into the importance of expansins, we screened different expansin genes in the carrot genome. According to the result of carrot genome blast, a total of 30 carrot expansin genes were identified. These expansin genes were named as *DcEXP1*-*DcEXP30* in the light of previous nomenclature (Kende et al., 2004). Genes corresponding to the two differentially expressed expansin proteins were annotated as *DcEXP20* (Accession No.: g13058) and *DcEXP22* (Accession No.: g63815), respectively. In order to explore evolutionary relationship among the carrot expansins, the neighbor-joining (NJ) method was used to construct a phylogenetic tree based on the entire sequences of *Arabidopsis* and carrot expansin proteins (Figure 6 and Supplementary Figure S3, Supplementary Material S1). All the DcEXPs were clustered into four subfamily: EXPA, EXPB, EXLA, and EXLB. The EXPA subfamily had the largest size, which consisted of 24 carrot expansins. However, EXLA and EXLB subfamily had only one member, respectively. Meanwhile, both the two identified differentially expressed expansin proteins belonged to the EXPB family. The length of the 30 expansins in carrot were 206–348 amino acids in length. The results of physicochemical property analysis showed that the Mw was within the range of 22.4–38.2 kDa. Their *pI* ranged from 5.3 to 10.2 (Supplementary Table S2, Supplementary Material S1). Excluded EXLB subfamily, most of the expansins had *pI* values above 7.0.

### Structural Analysis of Expansin Amino Acid Sequences in Carrot

The amino acid sequences of 30 expansins in carrot were aligned (Supplementary Figure S4, Supplementary Material S1). Amino acid sequence of proteins which belonged to the same subfamily had a much higher similarity. The aligned identity between DcEXP20 and DcEXP22 that both belong to EXPB subfamily was 74.6%. Most of the expansin proteins had a signal peptide of 16–30 amino acids. However, four proteins, DcEXP26, DcEXP29, DcEXP16, and DcEXP19 lacked a signal peptide (Supplementary Table S2, Supplementary Material S1). Followed by the signal peptide, several conserved amino acid residues were identified. In the EXPA and EXPB subfamily, all expansins contained a HFD motif, excluded DcEXP11 and DcEXP10 that contained a HFV and QFD motif instead, respectively. Generally, eight conserved cysteine (Cys) residues were identified both in DcEXPA and DcEXPB, and between DcEXLA and DcEXLB six. Meanwhile, 11, 21, and 14 glycine (Gly) were also conserved, respectively (Supplementary Figure S4, Supplementary Material S1).

Motifs in the proteins and their detailed motif sequences were set out schematically in Figure 6. Ten proteins in EXPA subfamily shared all ten motif components, and others lacked one or two motifs. Motifs in the other three subfamilies were significantly different from that in EXPA. Members in EXPB subfamily contained motif 4–7, except DcEXP19. However, EXLA and EXLB subfamily only have one or two motifs, respectively. Overall, motif 4 was present in all expansins.

### Transcriptome-Based Identification of Expansin Genes During the Carrot Root Growth

**FIGURE 7:**
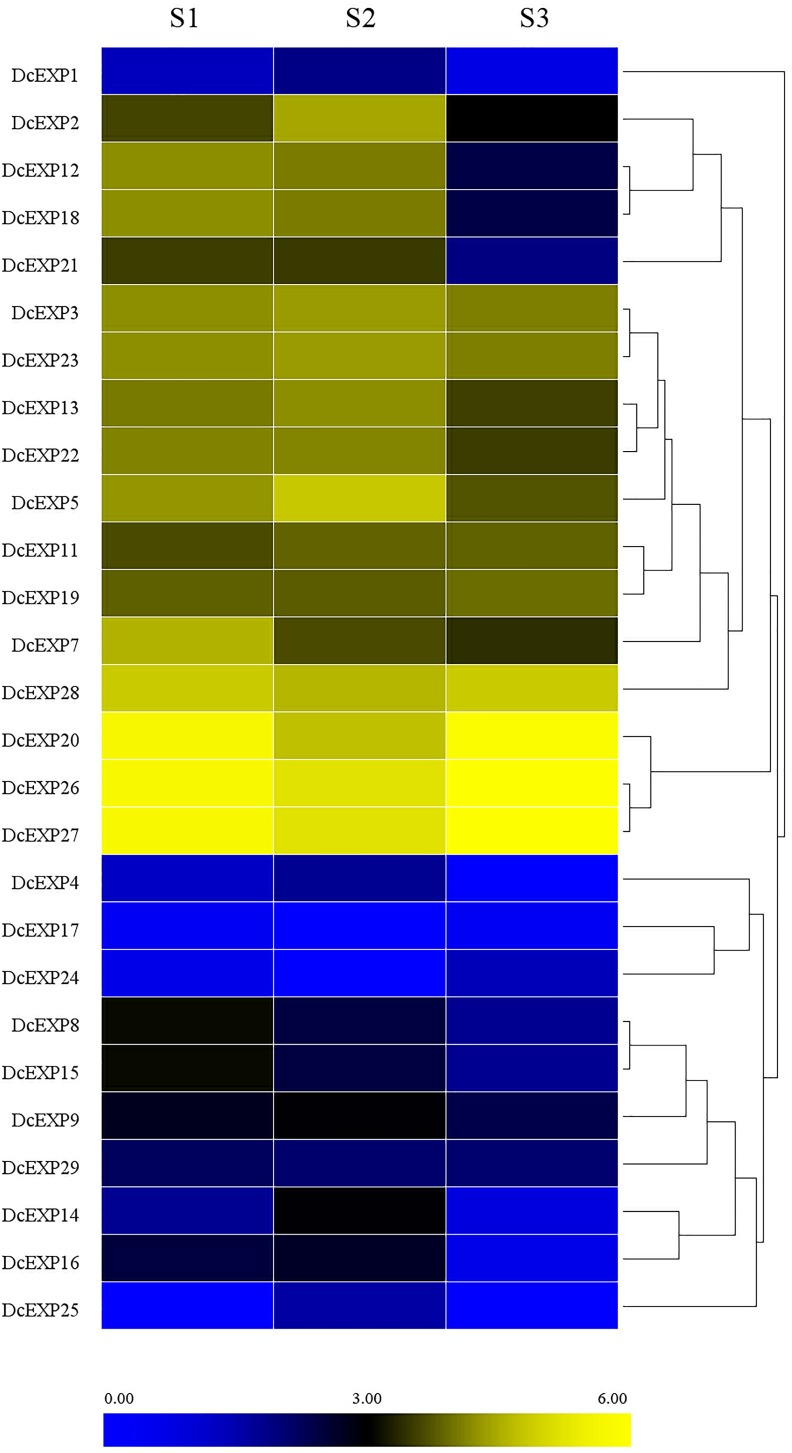
Expression profiles of carrot expansin genes.

With the aid of transcriptome data from preliminary study in our laboratory (Wang et al., 2015a), we probed into the transcript levels of the expansin genes during carrot root growth. A total of 26 expansin genes were screened through the transcriptome data. We drew a heat map on the basis of the per kilobase per million (RPKM) values to indicate their expression profiles (Figure 7). Almost half of the expansin genes had the highest expression at the second stage. However, we didn’t find the existence of *DcEXP17* and *DcEXP24* in S2. In contrast, *DcEXP25* only existed in S2 instead of S1and S3.

### Expression Profile Analysis of Expansin Genes During Carrot Root Development

**FIGURE 8:**
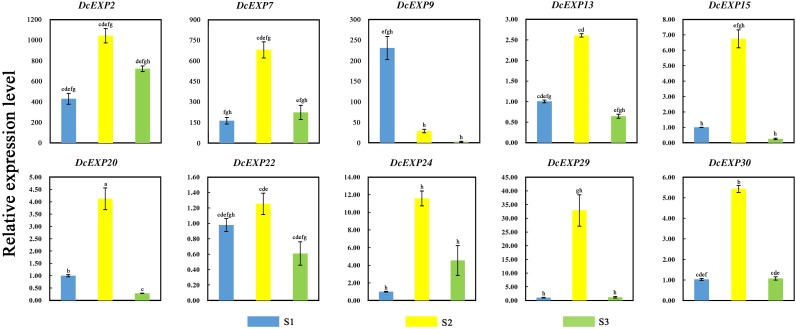
Relative expression levels of 10 carrot expansin genes. Different lowercase letters indicate significant differences at *P* < 0.05.

In order to investigate the molecular mechanism regulating carrot root development, ten different expansin genes including *DcEXP20* and *DcEXP22* were selected randomly from each expansin subfamily for expression analysis (Figure 8). Relative expression level of each gene was calculated based on the expression of *DcEXP24* at S1 that hold the lowest expression level. With the exception of *DcEXP9*, all expansin genes showed the highest expression levels at S2. Among these genes, the expression level of *DcEXP29* at S2 was more than 30 times as much as that at S1. At the mature stage, the expression profiles of the expansin genes were generally reduced, some were even lower than the first stage. The results of comparisons among different expansin genes showed that *DcEXP20*, *DcEXP30*, *DcEXP13*, and *DcEXP22* represented higher expression levels. Compared to the results of iTRAQ, two differentially expressed expansins, DcEXP20 and DcEXP22, exhibited a consistent relationship between the trends of mRNA expression and protein abundance. The correlation coefficient between expression level of *DcEXP20*, *DcEXP22* and other expansin genes was analyzed by Pearson analysis (Table 2). The expression profiles of *DcEXP20* and *DcEXP22* were positively correlated with eight and six expansin genes, respectively.

**Table 2:** Correlations between the expression levels of *DcEXP20*, *DcEXP22*, and other expansin genes.

		*DcEXP2*	*DcEXP7*	*DcEXP9*	*DcEXP13*	*DcEXP20*	*DcEXP22*	*DcEXP24*	*DcEXP15*	*DcEXP29*	*DcEXP30*
*DcEXP20*	Correlation coefficient	0.769^∗^	0.954^∗∗^	−0.239	0.988^∗∗^	1	0.857^∗∗^	0.860^∗∗^	0.983^∗∗^	0.939^∗∗^	0.979^∗∗^
	Sig.(2-tailed)	0.016	0.000	0.536	0.000	–	0.003	0.003	0.000	0.000	0.000
*DcEXP22*	Correlation coefficient	0.427	0.740^∗^	0.167	0.846^∗∗^	0.857^∗∗^	1	0.577	0.793^∗^	0.711^∗^	0.766^∗^
	Sig.(2-tailed)	0.251	0.023	0.668	0.004	0.003	–	0.104	0.011	0.032	0.016

*Correlations were determined by Pearson correlation coefficient analysis. ^∗^ indicates significance at P < 0.05. ^∗∗^ indicates significance at P < 0.01.*

## Discussion

Fleshy root is the most important economic organ of carrot (Wu et al., 2017). The growth and development of carrot is accompanied by the enlargement of its taproots, which is regulated by various factors (Perilli et al., 2012). Wang and his colleagues found that a total of 4,818 differentially expressed genes participated in the root expansion, of which some were related to hormones (Wang et al., 2015a). In later studies, multiple hormones were proved to be involved in the root development, such as cytokinins (Wang et al., 2015b) and gibberellins (Wang et al., 2015c). In this study, we found that during carrot root development, the vascular cambium split in different directions and then secondary xylem and secondary phloem came into being. This directly caused the enlargement of carrot roots. Moreover, at different stages, the rate of expansion was not constant which might be related to the protein or gene expression at different stages.

To further understand the dynamic changes in carrot root development, iTRAQ was used to investigate the proteomes of carrot roots at different developmental stages. Among the 2,845 proteins obtained, numerous proteins were expressed in all three stages which confirmed their important roles. Some of them were only expressed in one or two periods, this might be due to their stage specific characteristics. As a whole, 526 DEPs were identified during the whole developmental phase. These proteins could provide important resources for later research on the molecular mechanism of root development. By annotating the function of these DEPs, the results demonstrated that these proteins were involved in a variety of primary metabolisms, such as metabolism of energy, carbohydrate and amino acid. Cell division is the basis of plant growth and development which directly influences on the organ morphogenesis. This process is regulated by multiple factors, including cell division cycle proteins and cyclin-dependent kinases (Lipka et al., 2015). The high expression of these proteins in S2 and S3 might play essential roles in root development. As the hub of three major substance metabolisms (carbohydrates, lipids, and amino acid metabolisms), glycolysis and the TCA cycle provides a lot of necessary energy for plant growth (Huang et al., 2017; Jason et al., 2018). Our iTRAQ results showed that a large number of DEPs related to TCA cycle or energy metabolism, such as NADH dehydrogenase, UDP-glucose pyrophosphorylase and NADP-dependent malate dehydrogenase. These proteins were fully expressed during plant development to provide energy and substrate for the expansion of carrot root (Jha and Dubey, 2004; Yamori and Shikanai, 2016). We could also observe the defense system that was continuously built during the growth process. Defense-related proteins including heat shock proteins, pathogenesis-related proteins and catalase were abundant in fully developed roots. In the later stage of carrot root development, we also found some DEPs related to metabolism of glycan, some secondary metabolites and vitamins. This might mean that during the later period, some quality characters were constantly being built along with the development of the fleshy roots.

Among the identified DEPs, we noticed the expression changes of expansin proteins during carrot root development. Therefore, we further explored the expansin family in carrot. Expansins belong to a relatively conserved gene family which has a wide distribution in different plants (Mendre et al., 1992; Rose et al., 2000). With the completion of whole genome sequencing of various species, expansin gene families in a variety of plants have been identified in succession (Sampedro et al., 2005; Dal Santo et al., 2013; Zhu et al., 2014). There are 36 expansin genes in *Arabidopsis* genome, it is expected that there should be more expansin genes in carrots compared to *Arabidopsis*. However, based on the *Arabidopsis* expansin gene family (Sampedro and Cosgrove, 2005), only 30 *DcEXP* genes were found in the carrot genome. The results indicated that some of the duplicated expansin genes might be lost in the course of evolution. To understand the carrot expansin family in depth, we drew a phylogenetic tree which consisted of four subfamilies. Among them, the EXPA family had 24 members which were 60% of the whole family. Similar phenomenon also exists in some other species, such as soybean (Zhu et al., 2014) and rice (Sampedro and Cosgrove, 2005). There are more members belonging to EXPA subfamily of these plants. The results confirmed the conservation of expansin gene family in plants. In the process of gene family evolution, independent gene duplication may result in the retention of some highly conserved sequence and similar amino acid residue (Moore and Purugganan, 2003). Amino acid sequence alignment demonstrated that genes in the same expansin subfamily of carrot showed high motif conservation. However, there were certain differences in the gene structure among different sub-groups. We speculated that some motif additions or deletions might have occurred during the evolution of the carrot expansin gene family.

According to the expression analysis of expansins, we hypothesized that expansins might play important roles during the middle stage of carrot root development. With the maturation of fleshy roots, their expression was reduced gradually. The expression patterns of different genes were similar, suggesting that they might also work together to regulate root development. Research in other plants has also shown the relationship between expansins and plant growth. Previous work found that *LeEXP10* was abundantly expressed in the early stage of seed development in tomato. As the seeds matured, the expression level gradually decreased (Chen and Bradford, 2000). Experiment in soybean showed that at different stages of root development, the growth rates of primary and secondary roots were positively correlated with the expression of *GmEXP1*. During the rapid growth of soybean roots, the expression of *GmEXP1* was relatively high, opposite in the slow growth stage (Chen and Bradford, 2000). These results confirmed that expansins might play important roles during plant growth (Pien et al., 2001; Yoo et al., 2003). Previous studies showed that expansin protein is an important part of the plant cell wall (Li et al., 2002). By enzyme catalysis, expansin proteins can regulate plant growth by loosening cell wall components, stretching cells and enhancing cell flexibility (Rayle and Cleland, 1992).

**FIGURE 9:**
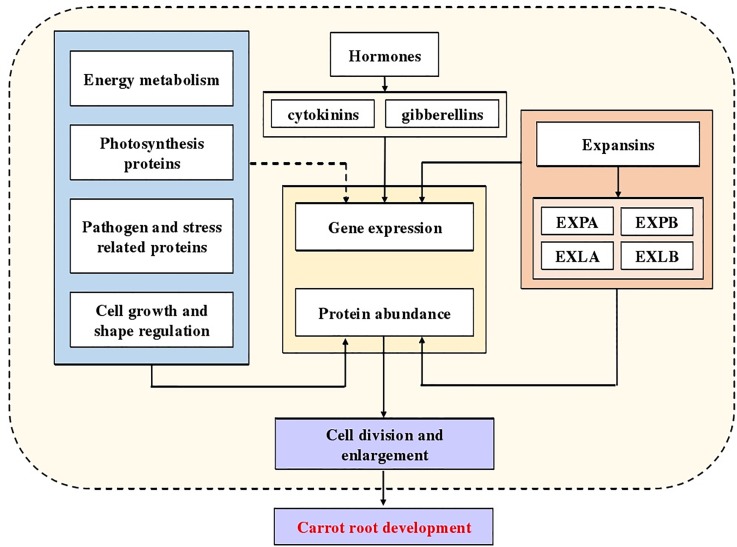
Potential molecular mechanism of carrot root development.

In our study, we identified numerous DEPs related to the molecular mechanism of carrot development, especially expansins. Combining the conclusions of predecessors, a diagram indicated the potential mechanism of carrot root development was summarized (Figure 9). Our results provide a new insight into further research, using mutants and transgenic technology to understand molecular mechanisms involved in carrot root development and possible role of expansins in this process.

## Data Availability

The datasets generated for this study can be found in GenBank, ProteomeXchange, MK190680; MK190681; and PXD011983.

## Author Contributions

A-SX and Y-HW conceived and designed the experiments. Y-HW, FQ, G-LW, J-NH and Z-SX performed the experiments. Y-HW, FQ, and TL analyzed the data. A-SX contributed reagents, materials, and analysis tools. Y-HW wrote the manuscript. A-SX and G-LW revised the manuscript. All authors read and approved the final manuscript.

## Conflict of Interest Statement

The authors declare that the research was conducted in the absence of any commercial or financial relationships that could be construed as a potential conflict of interest.

## Footnotes

1 http://proteomecentral.proteomexchange.org

2 https://www.arabidopsis.org/browse/genefamily/expansin.jsp

3 https://phytozome.jgi.doe.gov/pz/portal.html

4 http://supfam.org/SUPERFAMILY/index.html

5 http://www.biosoft.net/sms

6 http://www.cbs.dtu.dk/services/SignalP/

## Abbreviations

DAS: days after sowing
DEP: differentially expressed protein
DTT: dithiothreitol
EXLA: expansin-like A
EXLB: expansin-like B
EXPA: expansin A
EXPB: expansin B
FA: formic acid
FDR: false discovery rate
RPKM: per kilobase per million
RT-qPCR: quantitative reverse transcription-polymerase chain reaction
S1/S2/S3: stage 1/stage 2/stage 3
TCA: tricarboxylic acid
TEAB: triethylammonium bicarbonate.
