# Supplementary material for: iTRAQ-Based Quantitative Proteomics and Transcriptomics Provide Insights Into the Importance of Expansins During Root Development in Carrot
Source: Front Genet. 2019 Mar 29;10:247. doi: 10.3389/fgene.2019.00247 (PMC6449468; doi:10.3389/fgene.2019.00247)
Supplement: Supplementary file 1 [file Data_Sheet_1.PDF]

Frontiers in Genetics

iTRAQ-based quantitative proteomics and transcriptomics provide insights into the importance of expansins during root development in carrot

*Ya-Hui Wang[1,#], Feng Que[1,#], Guang-Long Wang[1, 2], Tong Li[1], Zhi-Sheng Xu[1] and Ai-Sheng Xiong[1, *]*

[1]State Key Laboratory of Crop Genetics and Germplasm Enhancement, College of Horticulture, Nanjing Agricultural University, Nanjing, Jiangsu 210095, China

[2]School of Life Science and Food Engineering, Huaiyin Institute of Technology, Huaian, Jiangsu 223001, China

----

Dr. Ai-Sheng Xiong

Professor

State Key Laboratory of Crop Genetics and Germplasm Enhancement,

College of Horticulture,

Nanjing Agricultural University,

1 Weigang, 210095, Nanjing, China

Telephone: +86-25-8439-6790 Fax: 86 25 84396790

Email: xiongaisheng@njau.edu.cn

Supplementary data

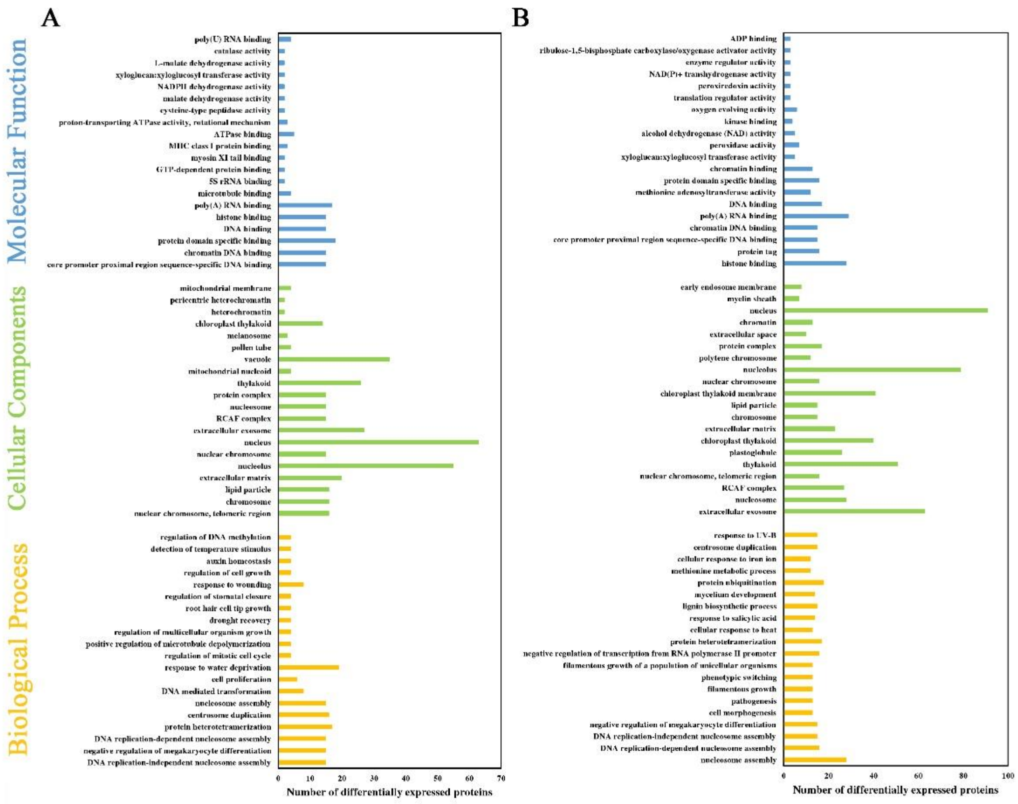

Fig. S1 GO classification of DEPs identified from carrot roots at different developmental stages. (A) GO classification of DEPs identified between S1 and S2. (B) GO classification of DEPs identified between S2 and S3. Top 20 terms of ‘biological process’, ‘cellular component’ and ‘molecular function’ were counted according to the *P* value in ascending order.

DcEXP20

```
1   ATGTATATCTTTCGCCAATTGCCACTCCTCCTCCTCTGTTTTTC
     M  Y  I  F  R  Q  L  P  L  L  L  L  C  F  F
46  ATTCCATTCTCCGCTGCAGCGCCTCTCCATCGAGGCGCCCCTGCC
     I  P  F  S  A  A  A  P  L  H  R  G  A  P  A
91  TCGCGTTGGTTCAGTGCCACTGCCACCTGGTACGGGAGCCCTGAC
     S  R  W  F  S  A  T  A  T  W  Y  G  S  P  D
136 GGCGATGGCAGTGATGGAGGTGCATGTGGGTACGGATCAATGGTG
     G  D  G  S  D  G  G  A  C  G  Y  G  S  M  V
181 GATGTGAAGCCATACAGGGCGAGAGTTGGCGCGGTGAGTCCGATT
     D  V  K  P  Y  R  A  R  V  G  A  V  S  P  I
226 TTGTTCAAAGGTGGTGAAGGTTGCGGCGCCTGTTACAAGGTCAGG
     L  F  K  G  G  E  G  C  G  A  C  Y  K  V  R
271 TGTTTGGACAGGTCTATTTGCGCCCGCAGAGCTGTCACTGTGATC
     C  L  D  R  S  I  C  A  R  R  A  V  T  V  I
316 ATCACTGATGAGTGCCCCGGGGGCTATTGCTCCGGGGGCCGCACA
     I  T  D  E  C  P  G  G  Y  C  S  G  G  R  T
361 CATTTTGACCTCAGCGGAGCTGCATTCGGCCGTTTGGCAGTCTCT
     H  F  D  L  S  G  A  A  F  G  R  L  A  V  S
406 GGTGAACGTAACCAGCTCCGCAACCGTGGTGTCATCCCTGTCACC
     G  E  R  N  Q  L  R  N  R  G  V  I  P  V  T
451 TACCGCAGGACAGCGTGTAAATATCCGGGGAAAAACATAGCGTTT
     Y  R  R  T  A  C  K  Y  P  G  K  N  I  A  F
496 AAGGTGAATGAAGGATCGGCTGATCATTGGCTTTCTCTTTTAGTT
     K  V  N  E  G  S  A  D  H  W  L  S  L  L  V
541 GAGTTTGAGGATGGTGATGGGGATCTTGGTTCCATGCATATTAAA
     E  F  E  D  G  D  G  D  L  G  S  M  H  I  K
586 CAGGCAGGATCAAGCGAGTGGCTAGAGATGAGTCACGTCTGGGGA
     Q  A  G  S  S  E  W  L  E  M  S  H  V  W  G
631 GCAACCTGGTGCAAAAATGGAGGACCGTTGAAAGGACCATTTTCA
     A  T  W  C  K  N  G  G  P  L  K  G  P  F  S
676 GTGAAAGTAACGACTCTGGCAACCGCTAAAACGCTATCTGCTAGA
     V  K  V  T  T  L  A  T  A  K  T  L  S  A  R
721 GATGTTATTCCAGCCAATTGGTCTCCGAAAGCCACTTACACCTCC
     D  V  I  P  A  N  W  S  P  K  A  T  Y  T  S
766 CGCCTTAATTTCTTCGTCTAG
     R  L  N  F  F  V  *
```

DcEXP22

```
1   ATGGTCAGCTTCACCCACCAATCTCTCGCGCCGCTTCTTTCCTTT
     M  V  S  F  T  H  Q  S  L  A  P  L  L  S  F
46  CTACTCCTGCTCCTACATTCCTTCTCCGCCGCCTCCTCCCCTCTC
     L  L  L  L  L  H  S  F  S  A  A  S  S  P  L
91  AAATCCGACCTACATTGGCGTCCCGCCACCGCCACTTGGTACGGC
     K  S  D  L  H  W  R  P  A  T  A  T  W  Y  G
136 AGCGCCGAAGGCGACGGCAGCGACGGTGGTGCATGCGGGTACGGA
     S  A  E  G  D  G  S  D  G  G  A  C  G  Y  G
181 TCGATGGTGGATGTGAAGCCGTTGAGAGCAAGAGTCGGAGCGGTG
     S  M  V  D  V  K  P  L  R  A  R  V  G  A  V
226 AGTCCAATTCTTTTCAAGGGAGGTGAGGGGTGTGGAGCATGTTAT
     S  P  I  L  F  K  G  G  E  G  C  G  A  C  Y
271 AAAGTCAGGTGTTTAGACAAGTCCATTTGTTCTAGAAGAGCTGTT
     K  V  R  C  L  D  K  S  I  C  S  R  R  A  V
316 ACGGTTATTATTACCGACGAGTGTCCCGGCGGTTACTGCTCCGGC
     T  V  I  I  T  D  E  C  P  G  G  Y  C  S  G
361 GGCCGGACGCATTTTGATCTCAGTGGAGCTGCATTTGGCCGCATG
     G  R  T  H  F  D  L  S  G  A  A  F  G  R  M
406 GCGATTACCGGTGAACACGGCCTGCTCCGTAATCGCGGCGAGATC
     A  I  T  G  E  H  G  L  L  R  N  R  G  E  I
451 TCTGTTATGTATCGCCGGACACCATGTAAATATCCTGGTAAAAAT
     S  V  M  Y  R  R  T  P  C  K  Y  P  G  K  N
496 GTGGCATTTCGTGTGAACGAAGGATCAACACCTTTCTGGCTATCT
     V  A  F  R  V  N  E  G  S  T  P  F  W  L  S
541 CTGTTGGTGGAGTTTGAGGACGGAGATGGAACGGTTGGATCTATG
     L  L  V  E  F  E  D  G  D  G  T  V  G  S  M
586 CACATTAGAGAGGCAGGGTCAACTGAGTGGTTAGAAATGAGTCAT
     H  I  R  E  A  G  S  T  E  W  L  E  M  S  H
631 TTGTGGGGAGCAAATTGGATCATCAATGGAGGACCATTAAAAGGA
     L  W  G  A  N  W  I  I  N  G  G  P  L  K  G
676 CCATTTTCAGTGAAGCTAACTTCACTCTCCACAGCAAGAACTCTC
     P  F  S  V  K  L  T  S  L  S  T  A  R  T  L
721 TCAGCCAGAGATGTCATTCCAAACAAATGGTCTCCAAAGGCTACT
     S  A  R  D  V  I  P  N  K  W  S  P  K  A  T
766 TACACCTCTCGCCTTAATTTCTAA
     Y  T  S  R  L  N  F  *
```

Fig. S2 Nucleotide acid and deduced amino acid sequences of DcEXP20 and DcEXP22.

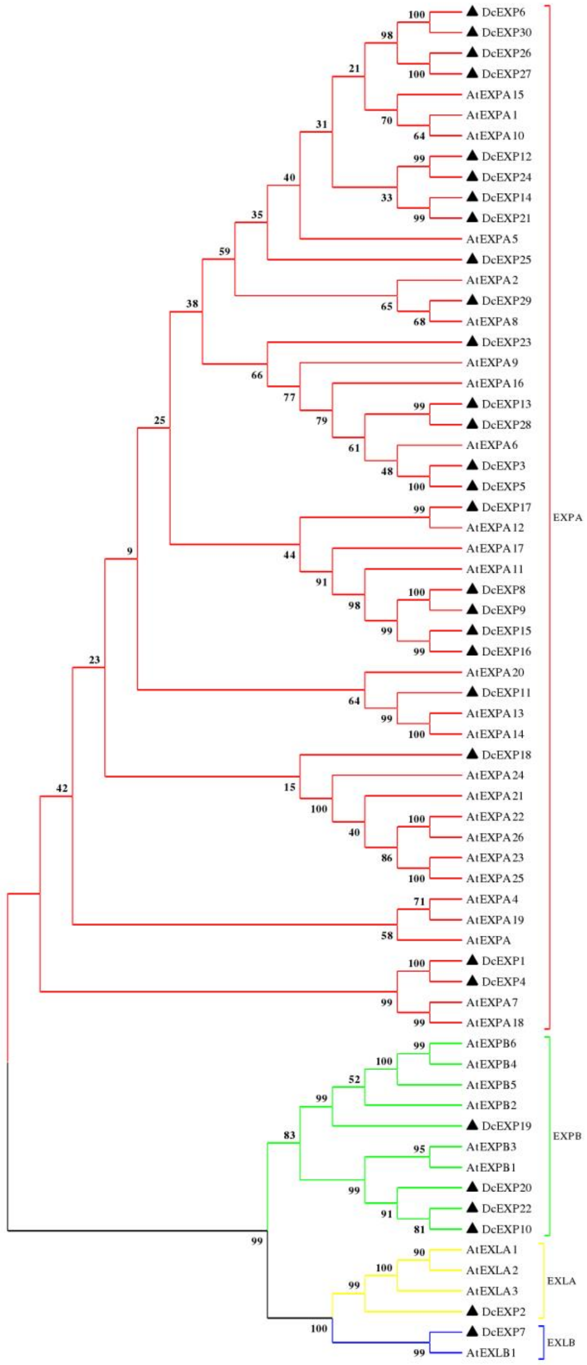

Fig. S3 Neighbor-joining phylogenetic tree of all the expansin proteins in carrot and *Arabidopsis*.

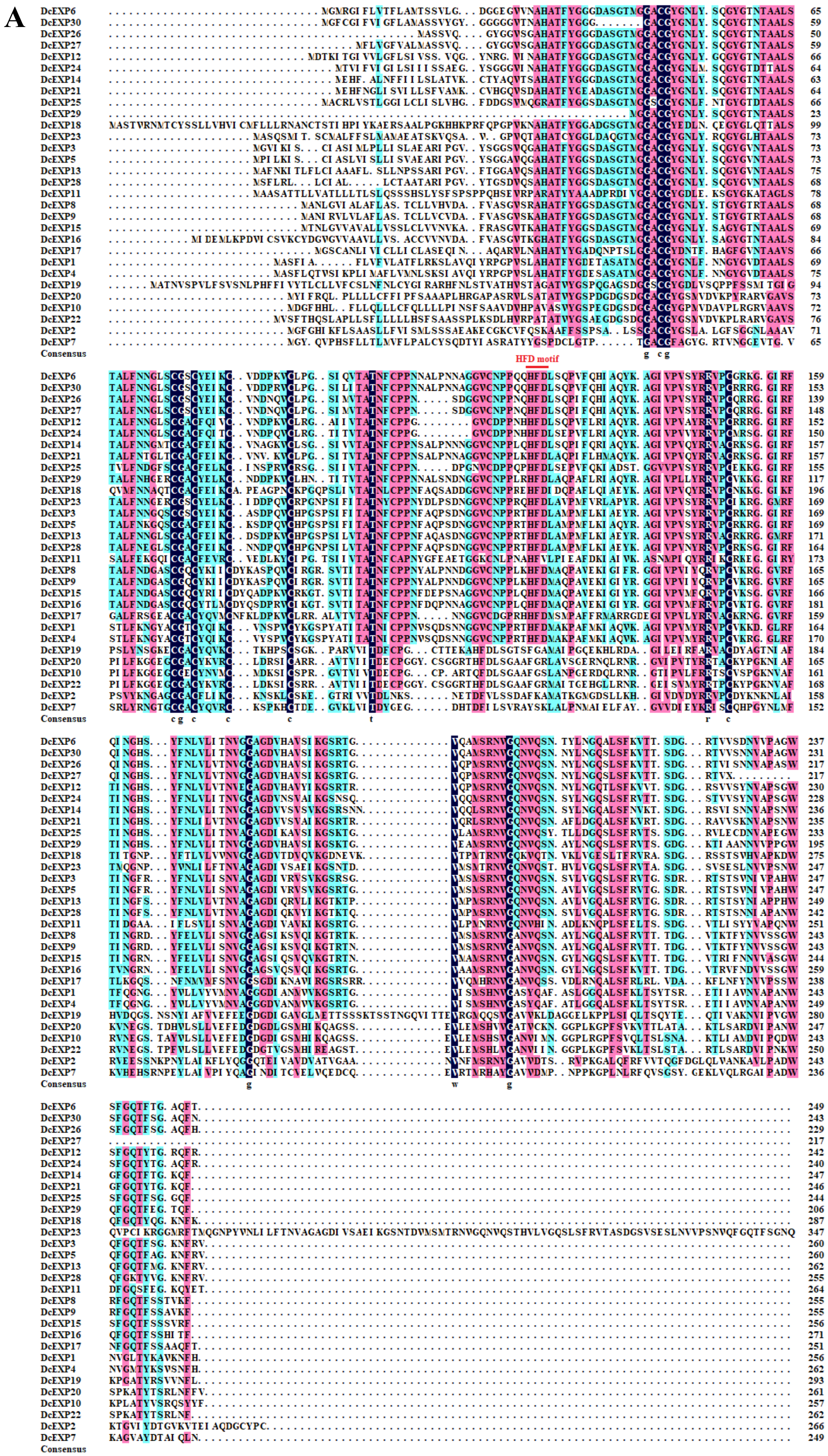
A
HFD motif
Consensus

B

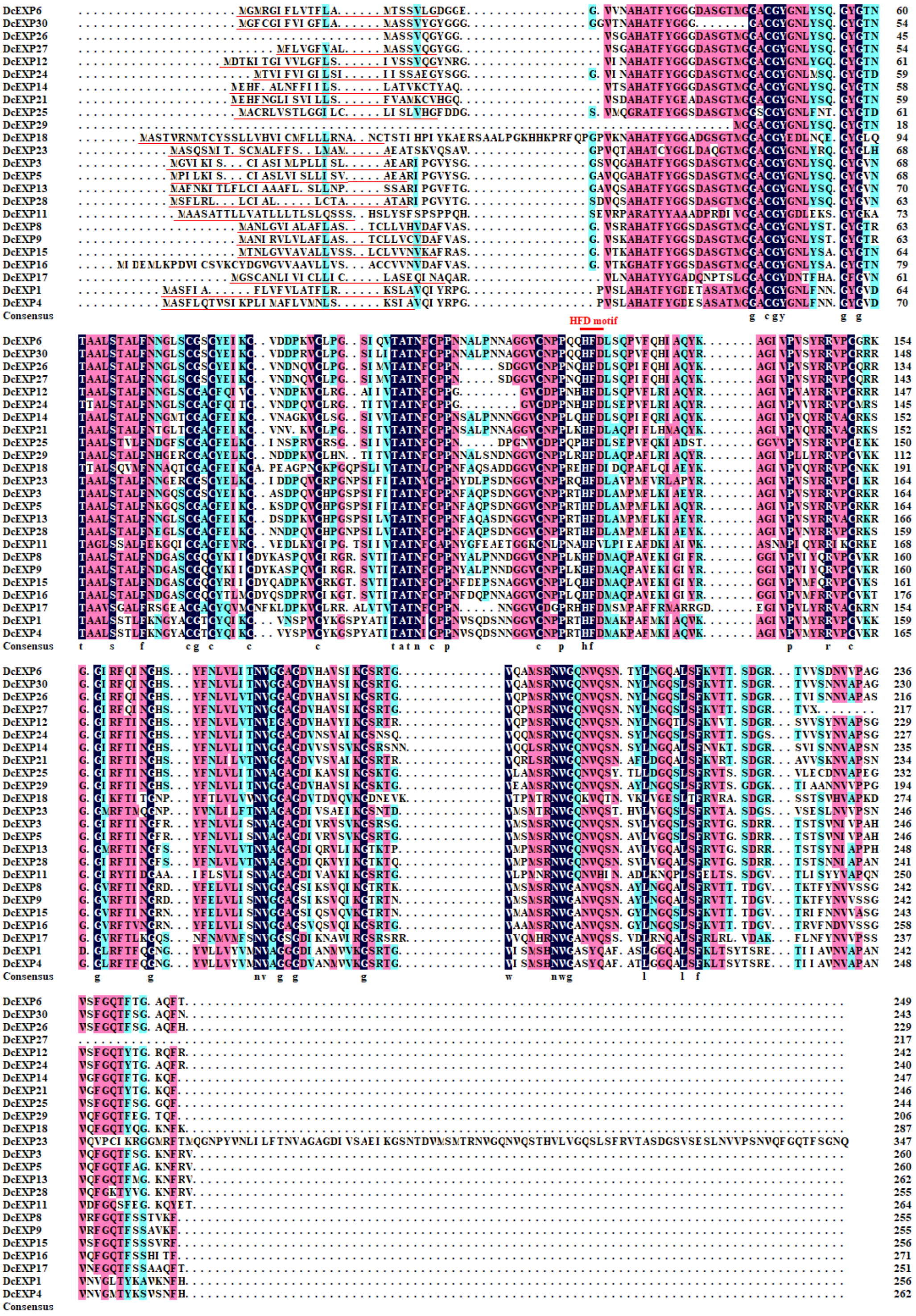

C

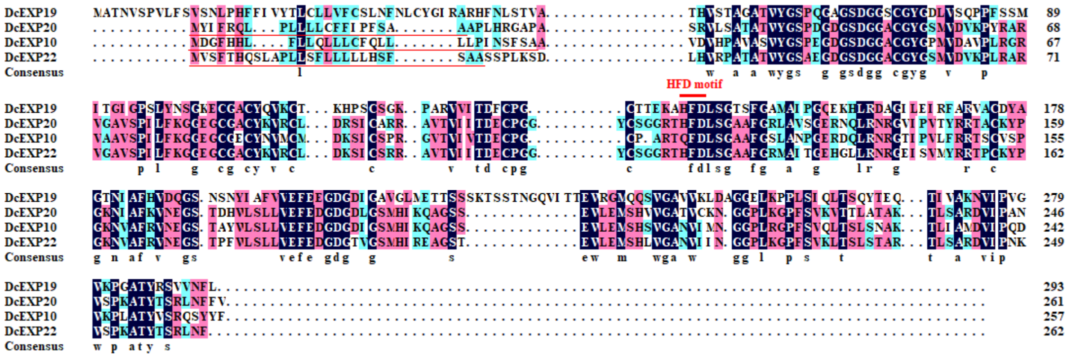

D

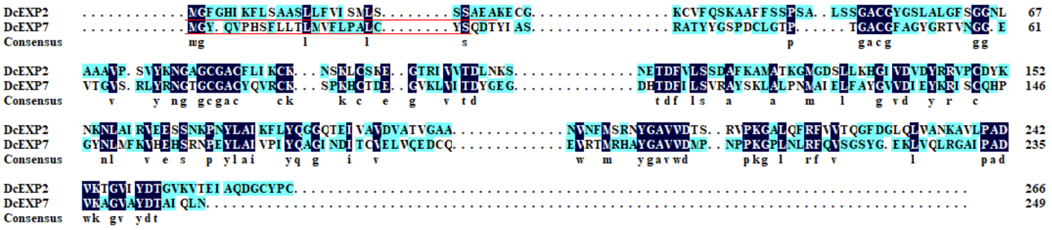

Fig. S4 Multiple sequence alignment and conserved amino acid residues of carrot expansins. (A) Multiple sequence alignment of all 30 carrot expansins. (B) Multiple sequence alignment of 24 DcEXPA proteins. (C) Multiple sequence alignment of 4 DcEXPB proteins. (D) Multiple sequence alignment of DcEXLA and DcEXLB proteins. Conserved amino acid residues are highlighted using different colors. The N-terminal signal peptides are underlined. The conserved His-Phe-Asp motif residues are indicated by HFD motif.

Table S1

Primer sequences used for cloning and qRT-PCR.

Gene	Purpose	Primer sequences(5’-3’)	
		Forward primer	Reverse primer
DcEXP20	clone	ATGTATATCTTTCGCCAATTGCC	CTAGACGAAGAAATTAAGGCGGG
DcEXP22		ATGGTCAGCTTCACCCACCAATC	TTAGAAATTAAGGCGAGAGGTGT
DcEXP2	RT-qPCR	GGTGCTGGTTGTGGTGCTTGTT	CCTTGGTCGCCATAGCCTTGAA
DcEXP7		AGCCAAGACACCTATATCGCCTCT	CACCACCATTGACAGTCCTTCCAT
DcEXP9		CAAACTTCTGCCCTCCAAACTATGC	ATCTAACTCCACCACGCTTAACACA
DcEXP13		ACCTTGCCATGCCCATGTTTCT	CCTTGATGAGGACTCGTTGGATGT
DcEXP15		ACATCTGTAACCATCACCGCAACAA	TGCCATCCAGTTCGTTCGTGTG
DcEXP20		ATTGCCACTCCTCCTCCTCTGTT	TTCACATCCACCATTGATCCGTACC
DcEXP22		ACTCCTGCTCCTACATTCCTTCTCC	TCAACGGCTTCACATCCACCATC
DcEXP24		CAATCACCGTCACCGCCACAA	GCTGTTGCTCTGCCAGTTCTGT
DcEXP29		GAGGTGCGGAGCTTGTTATGAGT	TTATGGATACGGCGTGGACATCTC
DcEXP30		GGTGGTGGTGGAGGTTGGACTAA	TAAGGCATTGTTTGGAGGGCAGAAG
DcActin		CGGTATTGTGTTGGACTCTGGTGAT	CAGCAAGGTCAAGACGGAGTATGG

Table S2 Characterization of expansin proteins in carrot.

Protein Name	Number of amino acid (aa)	Mw (kDa)	*pI*	signal peptide
DcEXP1	256	28.19	9.66	1-21
DcEXP10	257	27.8	5.25	1-28
DcEXP11	264	28.68	7.69	1-23
DcEXP12	242	26.36	9.68	1-22
DcEXP13	262	28.35	9.81	1-25
DcEXP14	247	26.57	9.29	1-22
DcEXP15	256	27.39	9.11	1-24
DcEXP16	271	29.06	7.79	NO
DcEXP17	251	27.87	9.80	1-23
DcEXP18	287	31.6	8.73	1-30
DcEXP19	293	31.37	6.83	NO
DcEXP2	266	28.4	8.67	1-27
DcEXP20	261	28.19	9.13	1-21
DcEXP21	246	26.54	9.47	1-23
DcEXP22	262	28.29	8.92	1-26
DcEXP23	348	38.15	8.04	1-23
DcEXP24	240	25.62	7.98	1-19
DcEXP25	244	26.07	6.70	1-21
DcEXP26	229	24.47	8.56	NO
DcEXP27	217	23.11	8.56	1-16
DcEXP28	255	27.68	9.28	1-18
DcEXP29	206	22.36	8.74	NO
DcEXP3	266	28.12	9.79	1-23
DcEXP30	243	25.96	8.94	1-20
DcEXP4	264	28.89	9.64	1-27
DcEXP5	260	28.12	10.21	1-23
DcEXP6	249	26.43	8.39	1-20
DcEXP7	249	27.65	6.84	1-23
DcEXP8	255	27.56	9.66	1-23
DcEXP9	255	27.6	9.52	1-23
